# Supplementary material for: Generation of False-Positive SARS-CoV-2 Antigen Results with Testing Conditions outside Manufacturer Recommendations: A Scientific Approach to Pandemic Misinformation
Source: Microbiol Spectr. 2021 Oct 20;9(2):e00683-21. doi: 10.1128/Spectrum.00683-21 (PMC8528119; doi:10.1128/Spectrum.00683-21)
Supplement: SUPPLEMENTAL FILE 1 — Supplemental material. Download Spectrum.00683-21-s0001.pdf, PDF file, 0.2 MB [file spectrum.00683-21-s0001.pdf]

**Table S1.** Impact of temperature and humidity on Panbio test performance.

| Temperature (°C) | | Results*** | | | | | | |
|---|---|---|---|---|---|---|---|---|
| | | | SARS-CoV-2 concentration (PFU/ml) | | | | | |
| **Pre-treatment*** | **Test Conditions**** | **PanBio Buffer** | **$1.2x10^5$** | **$1.2x10^4$** | **$5.0x10^3$** | **$2.5x10^3$** | **$1.25x10^3$** | **$1.1x10^3$** |
| **20** | **20** | 0/3 | 3/3 | 3/3 | 3/3 | 3/3 | 3/3 | 0/3 |
| | **4** | 0/3 | 3/3 | 3/3 | 3/3 | 3/3 | 3/3 | 0/3 |
| | **45** | 0/3 | 3/3 | 3/3 | 3/3 | 3/3 | 3/3 | 0/3 |
| **45** | **20** | 0/3 | 3/3 | 3/3 | 3/3 | 3/3 | 3/3 | 2/3 |
| | **4** | 0/3 | 3/3 | 3/3 | 3/3 | 3/3 | 3/3 | 1/3 |
| | **45** | 0/3 | 3/3 | 3/3 | 3/3 | 3/3 | 3/3 | 0/3 |
| **4** | **20** | 0/3 | 3/3 | 3/3 | 3/3 | 3/3 | 3/3 | 1/3 |
| | **4** | 0/3 | 3/3 | 3/3 | 3/3 | 3/3 | 3/3 | 1/3 |
| | **45** | 0/3 | 3/3 | 3/3 | 3/3 | 3/3 | 3/3 | 0/3 |
| **-20** | **20** | 0/3 | N/A | N/A | 3/3 | 3/3 | 3/3 | 0/3 |

*Pre-treatment was performed with test components in packaging.

**Samples stored at $45^oC$ were incubated at a relative humidity of 90-100%

*** Results are the summary obtained from triplicate testing.
